# Supplementary material for: Identification of Gene–Allele System Conferring Alkali-Tolerance at Seedling Stage in Northeast China Soybean Germplasm
Source: Int J Mol Sci. 2024 Mar 4;25(5):2963. doi: 10.3390/ijms25052963 (PMC10931751; doi:10.3390/ijms25052963)
Supplement: Supplementary file 1 [file ijms-25-02963-s001.zip › ijms-2820129-supplementary.pdf]

Supplementary figures and tables

Supplementary Figures

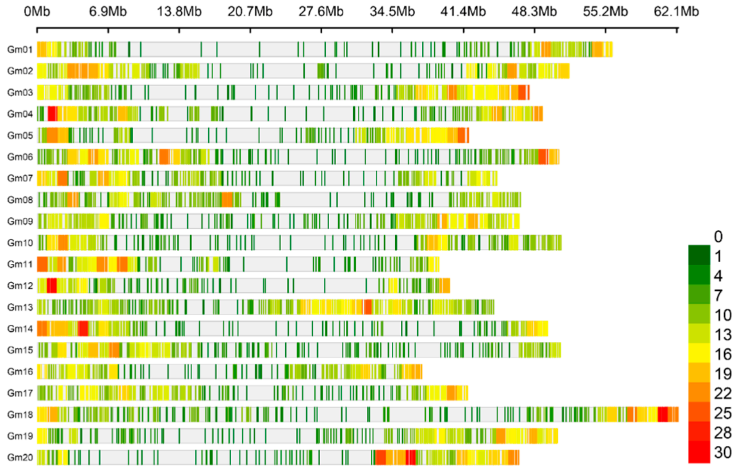

Figure S1 The distribution of GASMs on chromosomes in the NECSGP

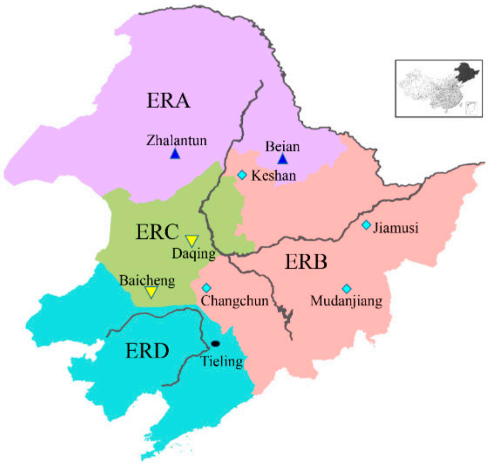

Figure S2 Schematic map of four ecological ecoregions in Northeast China

Note: ERA: Eco-region of Heilongjiang valleys, including northern part of Inner Mongolia, the purple area in the picture, represented by Beian and Zhalantun; ERB: Eco-region of Songhuajiang valleys, pink area, represented by Keshan, Jiamusi, Mudanjiang, and Changchun; ERC: Eco-region of Nengjiang valleys, grass green area, represented by Baicheng and Daqing; ERD: Eco-region of Liaohe valleys, light blue area, represented by Tieling. The dissemination path of soybeans in Northeast China was from ERD to ERB, then from ERB to ERA and ERC. The upper right corner shows the location of the Northeast region in China.

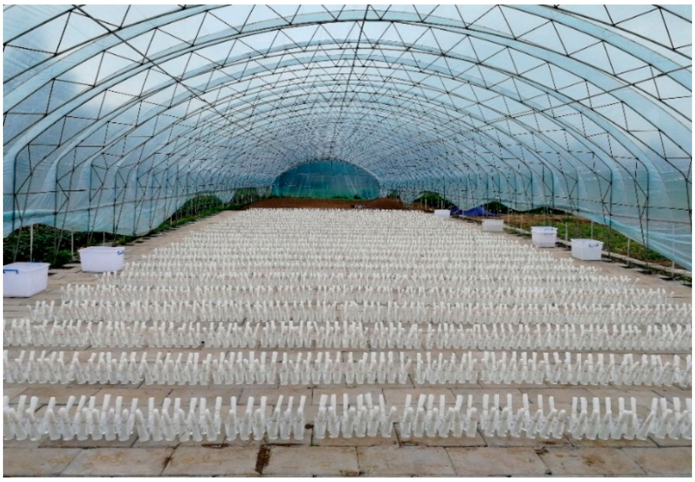

Figure S3 The test site for alkali-tolerance at seedling stage for the NECSGP

Note: The paper roll hydroponics was used to grow seedlings. A pair of stress and non-stress (control) cups were placed together. The stress treatment was a mixed solution based on 1/2 Hoagland's nutrient solution added with $NaHCO_3$: Na_2CO_3 (9:1) into a concentration of 220 mM with pH=9.8 while the control used 1/2 Hoagland's nutrient solution only.

Supplementary Tables

Table S1 Source and maturity groups of the tested 361 varieties in NECSGP

Maturity group	HLJ	JL	LN	IM	Foreign	Total
000	14			2		16
00	40			5		45
0	136	10	1	5	5	157
I	47	30	1	1		79
II	5	34	4			43
III	1	6	14			21
Total	243	80	20	13	5	361

Note: HLJ, Heilongjiang province; JL, Jilin province; LN, Liaoning province; IM, Inner Mongolia autonomous region.

Table S2 ANOVA of ATI under three environments of the NECSGP

Source of variation		*DF*	*MS*	*F* value	*P value*
Environment		2	0.0052	5.96	0.0026
Replication (Environment)		6	0.0018	2.08	0.0053
Genotype	ERA	60	0.0611	96.01	<0.0001
	ERB	229	0.0854	57.64	<0.0001
	ERC	7	0.1995	283.74	<0.0001
	ERD	61	0.1072	138.92	<0.0001
	All	360	0.0875	101.29	<0.0001
Genotype × Environment		720	0.0015	1.70	<0.0001
Error		2,148	0.0009		
Total		3,236			

Table S3 Allele structure of 10 top alkali-tolerant and 10 top alkali-sensitive varieties

Type	Code	Variety	Ecoregion	ATI	Positive allele		Negative allele	
					No.	Mean-effect	No.	Mean-effect
Tolerant	F284	Mufeng No. 3	ERB	0.86	69	1.88	63	-1.72
	F14	Beidou 14	ERB	0.86	71	1.76	61	-1.71
	F58	Mengdou 19	ERA	0.86	69	1.84	63	-1.70
	F337	Jilin 20	ERD	0.85	68	1.78	64	-1.64
	F243	Kenjian 35	ERB	0.85	66	1.77	66	-1.70
	F155	Mufeng No. 1	ERB	0.85	68	1.92	64	-1.60
	F81	Amsoy	ERB	0.84	71	1.82	61	-1.68
	F116	Kenfeng No. 10	ERB	0.84	76	1.90	56	-1.58
	F353	Dongnong No. 4	ERB	0.83	65	1.74	67	-1.67
	F154	Hefeng 22	ERB	0.83	71	1.88	61	-1.67
Average		10		0.85	69.4	1.83	62.6	-1.69
Sensitive	F124	Kenfeng 23	ERB	0.36	63	1.62	70	-1.92
	P183	Kangxian No. 7	ERC	0.37	61	1.59	71	-1.89
	F381	Tonghua Pingdingxiang	ERD	0.39	67	1.56	65	-1.74
	F334	Xiaojinhuang No. 9	ERD	0.41	66	1.66	66	-1.79
	F61	Mengdou 30	ERB	0.42	59	1.59	73	-1.94
	F45	Hefeng 35	ERB	0.44	70	1.57	62	-1.77
	F110	Changnong 20	ERB	0.45	63	1.67	69	-1.94
	F34	Changnong No. 5	ERD	0.45	63	1.69	69	-1.80
	F95	Jiyu 72	ERD	0.45	64	1.61	68	-1.85
	F136	Nenfeng 18	ERB	0.45	65	1.58	67	-1.75
Average		10		0.42	64.1	1.62	68.0	-1.84

Note: Ecoregion: Ecoregion in Northeast China; The dissemination path of soybeans in Northeast China was from ERD to ERB, then from ERB to ERA and ERC.

Table S4. The functional classifications of ATI genes identified in NECSP.

Gene code	Gene name	Main-effect R^2(%)	Biological process description	Gene function classification	Reported gene
gATI.1.1	*Glyma01g02580*	1.32	amino acid transport;	Material transport(II)	
gATI.1.2	*Glyma01g04515*	0.09	biological process	Biological process(VI)	
gATI.1.3	*Glyma01g22861*	0.19	NA	Unknown(VIII)	
gATI.1.4	*Glyma01g30320*	0.28	RNA processing;	Metabolic process (IV)	
gATI.1.5	*Glyma01g36070*	1.69	removal of superoxide radicals	Defense response(I)	
gATI.2.1	*Glyma02g00280*	0.05	NA	Unknown(VIII)	
gATI.2.2	*Glyma02g05640*	0.08	transmembrane receptor protein tyrosine kinase signaling pathway	Development(VII)	
gATI.2.3	*Glyma02g06730*	3.48	organ development	Development(VII)	
gATI.2.4	*Glyma02g08790*	0.03	multicellular organismal development	Development(VII)	
gATI.2.5	*Glyma02g09130*	0.70	NA	Unknown(VIII)	
gATI.2.6	*Glyma02g09240*	0.41	protein ubiquitination	Metabolic process (IV)	
gATI.2.7	*Glyma02g11151*	0.78	protein phosphorylation	Metabolic process (IV)	
gATI.2.8	*Glyma02g11335*	1.28	cell-cell signaling	Regulation of related (III)	
gATI.2.9	*Glyma02g14175*	0.12	spermine biosynthetic process	Biosynthetic process(V)	
gATI.2.10	*Glyma02g15470*	0.28	NA	Unknown(VIII)	
gATI.2.11	*Glyma02g16850*	0.39	biological process	Biological process(VI)	
gATI.2.12	*Glyma02g37010*	0.05	NA	Unknown(VIII)	
gATI.2.13	*Glyma02g38673*	0.19	NA	Unknown(VIII)	
gATI.2.14	*Glyma02g40220*	0.06	biological process	Biological process(VI)	
gATI.2.15	*Glyma02g44350*	0.24	acetyl-CoA metabolic process	Metabolic process (IV)	
gATI.3.1	*Glyma03g07890*	0.74	vacuole organization	Defense response(I)	
gATI.3.2	*Glyma03g27770*	2.23	oxidation-reduction process	Biological process(VI)	
gATI.3.3	*Glyma03g30270*	2.14	biological process	Metabolic process (IV)	*Glyma03g31691*(1.34Mb)
gATI.3.4	*Glyma03g36720*		hydrogen peroxide catabolic process	Defense response(I)	
gATI.3.5	*Glyma03g37221*	0.33	response to molecule of fungATI origin	Biological process(VI)	
gATI.4.1	*Glyma04g10720*	2.00	biological process	Biosynthetic process(V)	
gATI.4.2	*Glyma04g43300*	2.88	phosphatidylinositol biosynthetic process	Metabolic process (IV)	
gATI.5.1	*Glyma05g19630*	0.02	protein phosphorylation	Metabolic process (IV)	
gATI.5.2	*Glyma05g24760*	0.09	N-terminal protein myristoylation	Defense response(I)	
gATI.5.3	*Glyma05g27300*	0.94	defense response signaling pathway	Metabolic process (IV)	*GsGST14*(2.13Mb)
gATI.5.4	*Glyma05g27690*	0.2	SRP-dependent cotranslational protein targeting to membrane	Metabolic process (IV)	*GsGST14*(1.76Mb)
gATI.5.5	*Glyma05g32890*	0.81	protein phosphorylation	Metabolic process (IV)	
gATI.6.1	*Glyma06g01490*	0.49	protein phosphorylation	Defense response(I)	
gATI.6.2	*Glyma06g05300*	1.82	response to oxidative stress	Regulation of related (III)	
gATI.6.3	*Glyma06g07980*	4.66	positive regulation of transcription	Regulation of related (III)	
gATI.6.4	*Glyma06g17410*	0.08	RNA processing	Metabolic process (IV)	
gATI.6.5	*Glyma06g19756*	0.20	lipid metabolic process	Metabolic process (IV)	*GsBOR2*（579.6kb）
gATI.6.6	*Glyma06g47010*	0.14	NA	Unknown(VIII)	
gATI.7.1	*Glyma07g00400*	0.73	acetyl-CoA metabolic process	Metabolic process (IV)	
gATI.7.2	*Glyma07g05620*	2.45	DNA catabolic process	Metabolic process (IV)	
gATI.7.3	*Glyma07g06640*	0.10	lipid metabolic process	Biological process(VI)	
gATI.7.4	*Glyma07g07360*	0.12	biological process	Biological process(VI)	
gATI.7.5	*Glyma07g08290*		NA	Unknown(VIII)	
gATI.7.6	*Glyma07g10280*	0.08	biological process	Metabolic process (IV)	
gATI.7.7	*Glyma07g14234*	0.06	metabolic process	Biosynthetic process(V)	
gATI.7.8	*Glyma07g31130*	1.48	hydrogen peroxide biosynthetic process	Biological process(VI)	
gATI.7.9	*Glyma07g37810*	0.16	biological process	Defense response(I)	
gATI.7.10	*Glyma07g38180*	2.9	response to wounding	Biological process(VI)	
gATI.8.1	*Glyma08g04620*	0.18	protein dephosphorylation	Biological process(VI)	
gATI.8.2	*Glyma08g10001*	0.02	biological process	Biological process(VI)	
gATI.8.3	*Glyma08g18320*	0.38	biological process	Defense response(I)	*GsMIOX1a*(2.41Mb)
gATI.8.4	*Glyma08g42810*	0.05	biological process	Biological process(VI)	
gATI.8.5	*Glyma08g45301*	0.65	NA	Unknown(VIII)	
gATI.8.6	*Glyma08g45501*	0.13	NA	Unknown(VIII)	
gATI.8.7	*Glyma08g45610*	0.72	response to salt stress	Defense response(I)	
gATI.9.1	*Glyma09g12180*	0.03	biological process	Biological process(VI)	
gATI.9.2	*Glyma09g15860*	0.03	biological process	Biological process(VI)	
gATI.9.3	*Glyma09g33220*	2.52	transmembrane transport	Material transport(II)	
gATI.9.4	*Glyma09g40690*	0.68	metabolic process	Metabolic process (IV)	
gATI.9.5	*Glyma09g41821*	0.38	mRNA splicing, via spliceosome	Biological process(VI)	
gATI.10.1	*Glyma10g06480*	0.08	NA	Unknown(VIII)	
gATI.10.2	*Glyma10g06600*	0.80	defense response	Defense response(I)	
gATI.10.3	*Glyma10g07601*	1.32	DNA methylation	Biological process(VI)	
gATI.10.4	*Glyma10g14620*	0.06	NA	Unknown(VIII)	
gATI.10.5	*Glyma10g30100*	0.75	intra-Golgi vesicle-mediated transport	Material transport(II)	
gATI.10.6	*Glyma10g30320*	0.27	regulation of transcription, DNA-dependent	Biological process(VI)	
gATI.10.7	*Glyma10g31560*	1.32	ubiquitin-dependent protein catabolic process	Metabolic process (IV)	

gATI.10.8	*Glyma10g37420*	0.56	NA	Unknown(VIII)	*GsSLAH3*（573.5kb）
gATI.11.1	*Glyma11g01253*	0.05	response to osmotic stress	Defense response(I)	
gATI.11.2	*Glyma11g03580*	0.11	biological process	Biological process(VI)	
gATI.11.3	*Glyma11g07830*	0.71	signal transduction	Regulation of related (III)	*Gshdz4*(287.7kb)
gATI.11.4	*Glyma11g15140*	0.01	regulation of transcription,	Regulation of related (III)	
gATI.11.5	*Glyma11g27510*	0.10	regulation of transcription	Regulation of related (III)	
gATI.12.1	*Glyma12g03180*	0.10	DNA mediated transformation	Metabolic process (IV)	
gATI.12.2	*Glyma12g08010*	0.26	fatty acid biosynthetic process	Biosynthetic process(V)	
gATI.12.3	*Glyma12g30080*	0.24	salicylic acid biosynthetic process	Biosynthetic process(V)	
gATI.13.1	*Glyma13g00490*	0.38	biological process	Biological process(VI)	
gATI.13.2	*Glyma13g01900*	0.04	biological process	Biological process(VI)	
gATI.13.3	*Glyma13g23910*	0.64	histone acetylation	Regulation of related (III)	
gATI.13.4	*Glyma13g29225*	0.12	NA	Unknown(VIII)	
gATI.13.5	*Glyma13g29360*	0.45	biological process	Biological process(VI)	
gATI.13.6	*Glyma13g29520*	0.04	hyperosmotic salinity response	Defense response(I)	*GsNAC019*(5.09Mb)
gATI.13.7	*Glyma13g40690*	1.03	exocytosis	Defense response(I)	*GsNAC019*(3.68Mb)
gATI.13.8	*Glyma13g42650*	0.24	NA	Unknown(VIII)	
gATI.14.1	*Glyma14g04260*	0.14	hyperosmotic response	Defense response(I)	
gATI.14.2	*Glyma14g10780*	0.98	biological process	Biological process(VI)	
gATI.14.3	*Glyma14g20110*	0.31	endoplasmic reticulum unfolded protein response	Biological process(VI)	
gATI.14.4	*Glyma14g36130*	0.25	biological process	Biological process(VI)	
gATI.14.5	*Glyma14g37280*	2.84	biological process;	Development(VII)	
gATI.14.6	*Glyma14g38720*	0.05	photorespiration	Metabolic process (IV)	
gATI.15.1	*Glyma15g02310*	0.35	NA	Unknown(VIII)	
gATI.15.2	*Glyma15g04006*	0.32	transcription, DNA-dependent	Regulation of related (III)	
gATI.15.3	*Glyma15g07590*	0.33	embryo sac development	Regulation of related (III)	
gATI.15.4	*Glyma15g16830*	0.03	stomatal complex morphogenesis	Biological process(VI)	
gATI.15.5	*Glyma15g19900*		response to salt stress	Defense response(I)	*GsTIFY10a*(49.1kb)
gATI.15.6	*Glyma15g27480*	0.36	trehalose biosynthetic process	Biosynthetic process(V)	
gATI.15.7	*Glyma15g27750*	0.37	biological process	Biological process(VI)	
gATI.15.8	*Glyma15g32540*		translation	Regulation of related (III)	
gATI.16.1	*Glyma16g08960*	1.43	fatty acid beta-oxidation	Metabolic process (IV)	
gATI.16.2	*Glyma16g28270*	0.91	regulation of transcription	Regulation of related (III)	
gATI.16.3	*Glyma16g32650*	0.89	phloem or xylem histogenesis	Development(VII)	
gATI.16.4	*Glyma16g33831*	0.1	biological process	Biological process(VI)	
gATI.17.1	*Glyma17g07120*	1.66	toxin catabolic process	Metabolic process (IV)	*GsCHX19.3*(1.95Mb)
gATI.17.2	*Glyma17g08230*	0.95	cellular component organization	Development(VII)	
gATI.17.3	*Glyma17g10650*	0.39	biological process	Biological process(VI)	
gATI.17.4	*Glyma17g15720*	0.03	response to reactive oxygen species	Defense response(I)	
gATI.17.5	*Glyma17g16831*	0.11	response to salt stress	Defense response(I)	
gATI.17.6	*Glyma17g33020*	0.09	cell plate assembly	Development(VII)	
gATI.17.7	*Glyma17g33930*	2.56	actin cytoskeleton organization	Development(VII)	
gATI.17.8	*Glyma17g36130*	1.46	lipid metabolic process;	Metabolic process (IV)	
gATI.18.1	*Glyma18g01490*	2.71	hyperosmotic salinity response	Defense response(I)	*GsMIPS2*(814.6kb)
gATI.18.2	*Glyma18g03930*		hyperosmotic salinity response	Defense response(I)	*GsMIPS2*(1.10Mb)
gATI.18.3	*Glyma18g03975*	0.68	NA	Unknown(VIII)	
gATI.18.4	*Glyma18g04870*	0.35	biological process	Biological process(VI)	
gATI.18.5	*Glyma18g11512*	0.15	NA	Unknown(VIII)	
gATI.18.6	*Glyma18g15001*	0.03	NA	Unknown(VIII)	
gATI.18.7	*Glyma18g16761*	0.27	proteolysis	Metabolic process (IV)	
gATI.18.8	*Glyma18g16780*	0.71	defense response	Defense response(I)	
gATI.18.9	*Glyma18g28130*	3.00	carbohydrate metabolic process	Metabolic process (IV)	
gATI.18.10	*Glyma18g40780*	1.71	cysteine biosynthetic process	Biosynthetic process(V)	
gATI.18.11	*Glyma18g46101*	2.62	defense response	Defense response(I)	
gATI.18.12	*Glyma18g49450*	0.12	NA	Unknown(VIII)	
gATI.18.13	*Glyma18g50670*	0.17	response to salt stress	Defense response(I)	
gATI.18.14	*Glyma18g53285*	2.06	biological process	Biological process(VI)	
gATI.19.1	*Glyma19g31900*	1.28	response to chitin	Defense response(I)	
gATI.19.2	*Glyma19g35820*	0.78	biological process	Biological process(VI)	
gATI.19.3	*Glyma19g42710*		transport; transmembrane transport	Material transport(II)	
gATI.19.4	*Glyma19g43880*	0.06	defense response	Defense response(I)	
gATI.20.1	*Glyma20g01460*	0.22	response to salt stress	Defense response(I)	
gATI.20.2	*Glyma20g24740*	0.95	Mo-molybdopterin cofactor biosynthetic process	Biosynthetic process(V)	
gATI.20.3	*Glyma20g30360*	0.97	NA	Unknown(VIII)	
gATI.20.4	*Glyma20g30870*	0.17	histone methylation	Biological process(VI)	
Total	132	90.94			10

Note: Gene code: such as *gATI.1.1* where ATI means alkali tolerance, .1 represents Chromosome 1 and .1 represents its order on the chromosome according to its physical position. The position corresponds to the Williams 82 reference genome version 1 (Wm82.a1).

R^2: genetic contribution of a Gene.

Reported genes: the identified genes in this study are consistent to previously reported genes in SoyBase (http://www.soybase.org).

Table S5 Newly emerged/introduced positive ATI alleles in ERB of NECSP

Order	Allele code	Gene	Allele effect	Main-effect R^2(%)	In accessions and crosses	Gene ontology
1	*gATI.2.5.a3*	*Glyma02g09130*	0.007	0.70	√	Unknown (VIII)
2	*gATI.2.6.a3*	*Glyma02g09240*	0.022	0.41	NA	Protein ubiquitination(III)
**3**	*gATI.2.8.a4**	*Glyma02g11335*	0.085	1.28	NA	Cell-cell signaling(I)
4	*gATI.2.11.a3*	*Glyma02g16850*	0.026	0.39	√	Biological process(VI)
5	*gATI.4.1.a2**	*Glyma03g07890*	0.042	2.00	√	Biological process(VI)
6	*gATI.5.1.a2*	*Glyma05g19630*	0.010	0.02	√	Protein phosphorylation(III)
**7**	*gATI.7.7.a2**	*Glyma07g14234*	0.074	0.06	√	Metabolic process(IV)
8	*gATI.8.2.a3*	*Glyma08g10001*	0.027	0.02	NA	Biological process(VI)
9	*gATI.10.8.a2*	*Glyma10g37420*	0.045	0.56	NA	Unknown (VIII)
10	*gATI.11.3.a2*	*Glyma11g07830*	0.079	0.71	√	Signal transduction(I)
11	*gATI.11.5.a2*	*Glyma11g27510*	0.001	0.10	√	Regulation of transcription(III)
12	*gATI.12.3.a2*	*Glyma12g30080*	0.009	0.24	√	Salicylic acid biosynthetic process(V)
13	*gATI.13.1.a2*	*Glyma13g00490*	0.016	0.38	√	Biological process(VI)
14	*gATI.14.2.a4*	*Glyma14g10780*	0.010	0.98	NA	Biological process(VI)
15	*gATI.14.2.a7*	*Glyma14g10780*	0.016	0.98	NA	Biological process(VI)
16	*gATI.14.4.a2*	*Glyma14g36130*	0.034	0.25	√	Biological process(VI)
17	*gATI.15.5.a2*	*Glyma15g19900*	0.004	(0.03)	√	Response to salt stress(I)
18	*gATI.17.5.a3*	*Glyma17g16831*	0.013	0.11	NA	Response to salt stress(I)
19	*gATI.19.2.a2*	*Glyma19g35820*	0.051	0.78	√	Biological process(VI)
20	*gATI.20.3.a2*	*Glyma20g30360*	0.068	0.97	√	Unknown (VIII)
			0.004-0.085	0.002-2.00	13	

Note: In accessions and crosses: genes existed in the top ten accessions and top ten predicted crosses.
⋆ nominated important alleles. In R^2 column, the digit in parentheses is the R^2 of G×E.
